# Stabilizing Zinc Anodes with Water-Soluble Polymers as an Electrolyte Additive

**DOI:** 10.3390/ma18215040

**Published:** 2025-11-05

**Authors:** Xueyan Li, Xiaojiang Chen, Senlong Zhang, Jinrong Wang, Zhuo Chen, Yuexian Song

**Affiliations:** 1 Shanxi Key Laboratory of Catalysis and Energy Coupling, School of Chemical Engineering and Technology, Taiyuan University of Science and Technology, Taiyuan 030024, China; 2 School of Energy and Power Engineering, North University of China, Taiyuan 030051, China; 3 Physical Sciences and Engineering Division, King Abdullah University of Science and Technology (KAUST), Thuwal 23955-6900, Saudi Arabia; 4 Computer, Electrical and Mathematical Science and Engineering Division, King Abdullah University of Science and Technology (KAUST), Thuwal 23955-6900, Saudi Arabia

**Keywords:** zinc anodes, water-soluble polymers, additives, dendrite-free

## Abstract

**Highlights:**

**What are the main findings?**

- Different water-soluble polymers can be used as electrolyte additives.
- PAA exhibits stable cycling for more than 360 h at 5 mA cm^−2^, 2 mA h cm^−2^.

**What are the implications of the main findings?**

- PAA exhibits an optimum performance.
- PAA additive can inhibit hydrogen evolution, dendrite growth, and corrosion on the zinc anode.

**Abstract:**

Water-induced corrosion and zinc dendrite formation seriously disrupt the Zn plating/stripping process at the anode/electrolyte interface, which results in the instability of the Zn metal anode in aqueous zinc-ion batteries. To address the issues of the zinc metal anode, three water-soluble polymers with different hydrophilic groups—polyacrylic acid (PAA), polyacrylamide (PAM), and polyethylene glycol (PEG)—were designed as electrolyte additives in ZnSO_4_ electrolytes. Among them, the PAA-based system exhibited an optimal electrochemical performance, achieving a stable cycling for more than 360 h at a current density of 5 mA cm^−2^ with an areal capacity of 2 mA h cm^−2^. This improvement could be attributed to its carboxyl groups, which effectively suppresses zinc dendrite growth, electrode corrosion, and side reactions, thereby enhancing the cycling performance of zinc-ion batteries. This work provides a reference for the optimization of zinc anodes in aqueous zinc-ion batteries.

## 1. Introduction

With the growing focus on global climate anomalies and energy security, there is an urgent need to explore new types of sustainable energy sources to address issues such as energy crisis, air pollution, and greenhouse gas emissions [1,2,3]. Aqueous zinc-ion batteries (AZIBs) utilize aqueous electrolytes, featuring properties of high ionic conductivity, low cost, and non-flammability. Additionally, the zinc anode has a high theoretical capacity of up to 820 mA h g^−1^ and a low redox potential (−0.762 V vs. SHE), enabling the realization of high energy density and safe rechargeable batteries for multi-field applications [4,5]. However, AZIBs still face significant challenges in practical applications, which are primarily attributed to low Zn utilization, low Coulombic efficiency, and limited cycle life [6]. These drawbacks stem from three key issues: short circuits caused by Zn dendrite penetration through the separator, corrosion, and the hydrogen evolution reaction (HER) of Zn anode induced by water [7].

Electrolyte additive engineering has emerged as an effective strategy for enhancing the performance of AZIBs, which can mainly be attributed to its simple preparation process and low cost [8,9]. Typically, introducing additives into aqueous zinc-salt electrolytes will effectively stabilize the Zn anode via multiple mechanisms involving the in situ construction of an adaptable solid electrolyte interphase, modifying the solvated Zn^2+^ structure, and regulating Zn deposition orientation [10,11]. Multiple approaches, such as the use of ionic liquids, surfactants, and supramolecular materials, have been developed to enable highly reversible zinc anodes [12,13,14]. Among the numerous additives, water-soluble polymers have garnered extensive attention due to their remarkable efficacy in enhancing the interfacial stability and kinetics of zinc anodes [15,16,17]. The long-chain structure of water-soluble polymers can be adsorbed onto the surface of zinc anodes, forming a dynamic and dense polymer interfacial film. Their steric hindrance effect can restrict the local aggregation of Zn^2+^ ions on the electrode surface, thereby preventing the consequent initiation of dendrite growth [18,19,20,21,22]. Meanwhile, the strong hydrophilicity of hydrophilic groups reduces the activity of water molecules at the interface, minimizes the direct contact between water molecules and zinc metal, and thus inhibits hydrogen evolution reaction and corrosion [23,24,25,26,27]. Although water-soluble polymers have been employed as additives in aqueous electrolytes for Zn metal anodes, their research in the field of AZBs remains relatively superficial and unsystematic. In general, the hydrophilicity of water-soluble polymers is endowed by hydrophilic groups. However, the effects of different water-soluble polymer additives on the performance of AZIBs have rarely been investigated, especially the influence of different hydrophilic groups on the reversibility of Zn plating/stripping and the interfacial reaction mechanism.

In this work, three polymers containing distinct hydrophilic groups, namely polyacrylic acid (PAA), polyacrylamide (PAM), and polyethylene glycol (PEG) were selected as electrolyte additives to investigate their effects and mechanisms on the cycling stability of Zn metal anodes. It demonstrates that the Zn||Zn symmetric battery assembled with the PAA-based electrolyte exhibits an optimum performance, delivering a stable cycling lifespan of more than 360 h at 5 mA cm^−2^, 2 mA h cm^−2^, which is ascribed to its effective suppression of hydrogen evolution, dendrite growth, and corrosion on the Zn anode.

## 2. Materials and Methods

### 2.1. Materials Preparation

Zinc sulfate (ZnSO_4_·7H_2_O), polyacrylic acid, polyacrylamide, and polyethylene glycol were purchased from Shanghai Aladdin Biochemical Technology Co., Ltd. (Shanghai, China). The 1 mol L^−1^ ZnSO_4_ aqueous solution was prepared as the baseline electrolyte. PAA, PAM, and PEG were separately dissolved into this solution to obtain 1 mol L^−1^ ZnSO_4_ containing 0.5 g L^−1^ respective water-soluble polymers. Zn foils were punched into circular disks with diameters of 12 and 16 mm, respectively. The disks were ultrasonically cleaned in deionized water and anhydrous ethanol for 10 min each (three cycles), followed by drying in an oven for 1 h.

### 2.2. Electrochemical Performance Measurements and Characterization

Symmetric Zn||Zn batteries were assembled in CR2032 coin-type configurations. Galvanostatic cycling tests of the Zn||Zn symmetric batteries were conducted on a LAND battery testing system (CT3001A) at room temperature to evaluate the cycling stability of different electrolyte systems. Cu||Zn coin cells were prepared for exploring the zinc deposition behavior in copper substrate and testing the CE. Linear scanning voltammetry (LSV), electrochemical impedance spectroscopy (EIS), and Tafel plot measurements were carried out using an electrochemical workstation of Shanghai Chenhua Instrument Co., Ltd. (CHI660E, Shanghai, China). The LSV was tested with a scan rate of 10 mV s^−1^. The Tafel plot test was collected at a potential ranging from −0.90 V to 1.15 V at 1 mV s^−1^. EIS was measured within a range of 10^6^ Hz to 0.05 Hz. The LSV and EIS measurements were performed in a three-electrode system (working electrode of Zn, counter electrode of Pt, and reference electrode of Ag/AgCl with saturated KCl solutions). Surface morphology of the Zn anode was examined by scanning electron microscopy (SEM, JSM-7800F, JEOL, Tokyo, Japan). Raman spectra were obtained by Scientific LabRAM HR Evolution of HORIBA, Ltd. (Shanghai, China). Fourier transform infrared (FTIR) spectra were obtained by FTIR spectrometer (Thermo Scientific Nicolet iS5 of Thermo Fisher Scientific Inc., Waltham, MA, USA) to collect the vibration or stretch of the functional groups in the samples.

## 3. Results and Discussion

**Figure 1:**
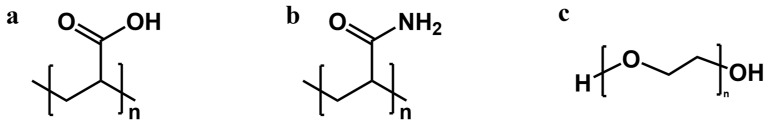
The molecular structure of (**a**) PAA, (**b**) PAM, and (**c**) PEG.

To investigate the effect of water-soluble polymer additives on the cycling stability of zinc anodes, PAA with a carboxyl group, PAM with amino groups, and PEG with hydroxyl groups (Figure 1) were respectively employed in 1 M ZnSO_4_ electrolytes as additives. The concentration of PAA, PAM, and PEG additives was 0.5 g L^−1^. Galvanostatic charge–discharge tests were conducted to obtain the cycling time of Zn||Zn symmetric battery with three polymer additives.

**Figure 2:**
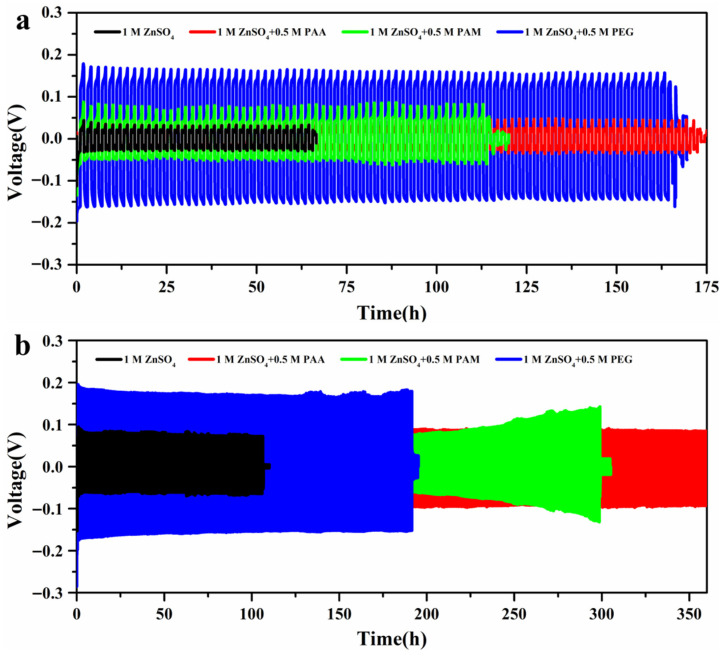
Galvanostatic Zn plating/stripping of long-term cyclic stability of Zn||Zn symmetric batteries at (**a**) 2 mA cm^−2^, 2 mA h cm^−2^, and (**b**) 5 mA cm^−2^, 2 mA h cm^−2^ with different electrolyte systems.

As shown in Figure 2a and Table 1, at a current density of 2 mA cm^−2^ and an areal capacity of 2 mA h cm^−2^, the battery with the PAA-based electrolyte exhibited the optimal cycle life of 172 h. The battery containing PAM and PEG additives also achieved a stable cycling for 114 h and 166 h, respectively. In contrast, the Zn||Zn cell assembled with the pure ZnSO_4_ electrolyte showed poor performance, suffering a short circuit after only 65 h, which could be attributed to Zn-dendrite formation. The comparative results of different electrolyte systems indicate that all three water-soluble polymer additives may effectively improve the cycling stability and reversibility of Zn anode.

To ascertain the effectiveness and universality of water-soluble polymer additives in modifying Zn anode, the cycling performance tests of Zn||Zn symmetric cells were carried out at a higher current density of 5 mA cm^−2^ and an areal capacity of 2 mA h cm^−2^ (Figure 2b). The PAA additive still demonstrated the optimal cycle life over 360 h, while the Zn||Zn symmetric battery with the ZnSO_4_ electrolyte exhibited a short circuit at 107 h due to the unstable interface of the Zn anode/electrolyte. When PAM and PEG were added, the cells had a stable cycling for 300 h and 192 h, respectively. This indicates that all three water-soluble polymer additives can effectively enhance the cycling stability of zinc anodes, confirming the universal effectiveness of such additives in improving the cycling performance of Zn symmetric batteries. In addition, especially compared with PEG-based systems, the PAA-modified electrolyte delivers a lower voltage hysteresis, which can be attributed to the facile desolvation processes of Zn^2+^ ions due to the strong interaction between the highly electronegative carbonyl groups and Zn^2+^ [28,29]. The CE was further obtained to evaluate the reversibility of Zn plating/stripping behavior in Figure S1. The Cu||Zn cell with ZnSO_4_ electrolyte exhibited a less than 90% CE before 40 cycles, and then had an abrupt drop, confirming a short circuit of battery. When PAA or PAM additives were added into the ZnSO_4_ solutions, the cells exhibited an average CE of 99.0% and 98.7%, respectively. However, the cycling life was relatively reduced following addition of PEG into ZnSO_4_ for 55 cycles. As a result, this confirms that the presence of PAA additive can significantly modify ZnSO_4_ electrolyte and enhance the cycling stability of Zn anode.

**Figure 3:**
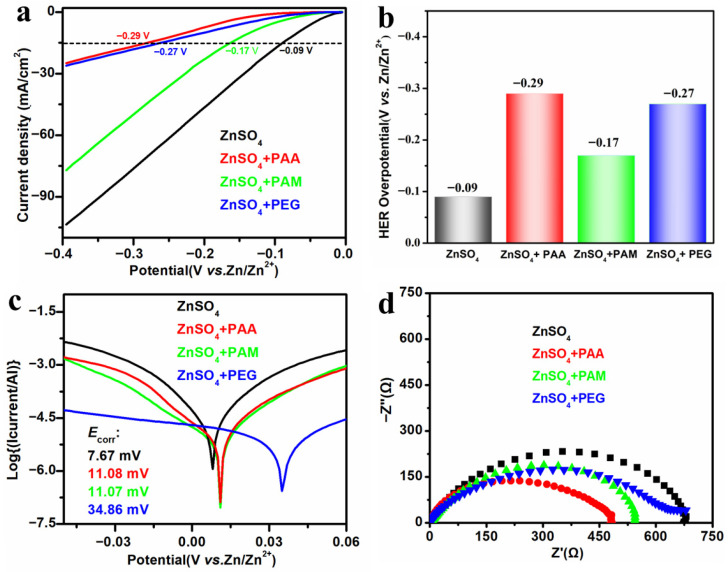
(**a**) LSV curves, (**b**) the overpotential of HER obtained at a current density of −15 mA cm^−2^ from LSV curves, (**c**) Tafel plots, and (**d**) Electrochemical impedance spectra of various electrolytes.

To investigate the mechanism of water-soluble polymer additives on the stable cycling performance of zinc anodes, the hydrogen evolution reaction was first studied. LSV measurements were performed on Zn||Zn symmetric batteries assembled with four electrolytes (containing PAA, PAM, and PEG additives, and pure ZnSO_4_ solution, respectively) (Figure 3a). It was found that at a fixed current density of −15 mA cm^−2^, the initial hydrogen evolution overpotential with PAA-based electrolytes was −0.29 V, which was lower than that of the electrolyte with the PEG additive (−0.27 V), the electrolyte with the PAM additive (−0.17 V), and the blank zinc sulfate electrolyte (−0.09 V), as summarized in Figure 3b. Clearly, the hydrogen evolution overpotentials of all three polymer electrolytes exhibited a significant negative shift, indicating that the presence of these polymers could mitigate the H_2_O-induced reaction rates, and PAA additive shows the strongest inhibitory ability. This could be ascribed to polymer additives that adsorbed at Zn anode to change the electrical double-layer structure [16,30].

To explore the electrochemical corrosion behavior of the metallic zinc anode with PAA, PAM, and PEG additives, Tafel polarization curves were carried out in pure ZnSO_4_ solutions and the electrolyte systems containing the three additives, respectively. As shown in Figure 3c, the corrosion potential of zinc anode in the three additive-containing solutions all showed a positive shift compared with that in the pure ZnSO_4_ solutions. This confirms that three water-soluble polymer additives can protect the metallic zinc anode, reduce the negative effects caused by corrosion reactions, and further improve the electrochemical performance of the metallic zinc anode.

To examine the effects of PAA, PAM, and PEG additives on the interfacial reaction kinetics of metallic zinc anode, electrochemical impedance spectroscopy measurements were conducted on Zn||Zn symmetric batteries. The results indicate that the charge transfer resistances in the electrolytes containing PAA, PAM, and PEG were all smaller than that in pure ZnSO_4_ electrolytes, with the PAA-containing system exhibiting the smallest resistance (Figure 3d). This result demonstrates that all three water-soluble polymer additives can promote the kinetics of zinc ions, and PAA especially displays the most excellent ability.

**Figure 4:**
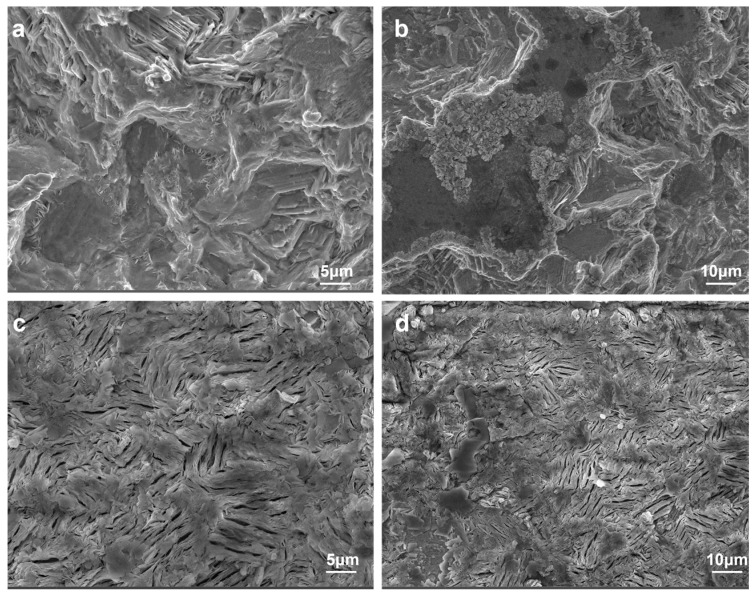
SEM images of Zn electrode in Zn||Zn symmetrical batteries after cycling in (**a**,**b**) 1 M ZnSO_4_ and (**c**,**d**) 1 M ZnSO_4_ with 0.5 g L^−1^ PAA electrolytes.

To investigate the effect of PAA additive on Zn deposition of metallic zinc anode, the metallic Zn anode was retrieved after 20 cycles under the conditions of 2 mA cm^−2^ and 2 mA h cm^−2^. Subsequently, the surface morphology of the cycled metallic Zn anode was characterized using an SEM. As shown in Figure 4a,b, a large number of protruding dendrite-like Zn deposits were observed on the surface of the Zn anode in pure ZnSO_4_ electrolytes. In contrast, the surface of the Zn electrode with PAA electrolyte was relatively flat and free of protruding Zn dendrites, as presented in Figure 4c,d. Furthermore, FTIR characterization was conducted to explore the species of Zn electrode following the immersion into ZnSO_4_ electrolytes with/without PAA additives (Figure S2). With PAA, peaks at 1583 cm^−2^ (C-O bond), 2850/2924 cm^−2^ (C-H bond), and 580 cm^−2^ (Zn-O bond) on the Zn anode were observed, indicating that PAA adsorbs on Zn electrode to construct a shielding layer, which could effectively manipulate uniform Zn nucleation. However, with pure ZnSO_4_, a wide O-H peak is visible, demonstrating the severe H_2_O-related by-products formation. This demonstrates that the presence of PAA additives could effectively suppress the formation of zinc dendrites and side reactions. Similarly, SEM images show that the Zn anode obtained with PAM and PEG electrolytes also shows a plat and smooth morphology (Figure S3).

In summary, the added water-soluble polymer additives (PAA, PAM, and PEG) can adsorb at the electrolyte/zinc anode interface and change the electrical double layer structure, thereby enhancing the Zn plating/stripping behavior and long-term stability of AZBs. Compared with PAM and PEG, the PAA additive is more effective in protecting the zinc metal anode and mitigating the effects caused by corrosion, HER, and zinc dendrite growth, thus improving the electrochemical performance of the zinc anode. This superiority may be attributed to the fact that the -COOH groups of PAA are more prone to forming strong coordination interactions with Zn^2+^ ions compared to the -NH_2_ groups of PAM and -OH groups of PEG [31,32,33]. Raman spectroscopy was further conducted to explore the Zn^2+^ coordination environment of different electrolytes, as shown in Figure S4. With PAA, an obvious peak was observed at 403 cm^−1^, and a new peak at 311 cm^−1^ was visible, indicating a direct interaction between Zn^2+^ and COOH/COO-. Such coordination can reconstruct the solvation structure of Zn^2+^ ions, facilitating the formation of a stable continuous Zn electrode/electrolyte interface [34,35]. However, with the other three electrolytes, the same FTIR spectra show that the addition of PAM/PEG cannot affect Zn^2+^ solvation structure. Meanwhile, PAA can immobilize free water molecules via its -COOH groups, reducing the activity of water in the electrolytes. This not only inhibits HER and zinc corrosion to improve zinc utilization efficiency but also significantly reduces the formation of the by-product zinc hydroxide sulfate, thereby extending the cycling lifespan and enhancing cycling stability in Zn-based batteries.

## 4. Conclusions

In this study, the effect of water-soluble polymers as electrolyte additives on the performance of zinc anodes was systematically investigated. The results revealed that the incorporation of water-soluble polymers (PAA, PAM, and PEG) significantly enhances the stability of zinc anodes, and that among the polymers, PAA exhibits the optimal cycling stability. Specifically, PAA enables Zn||Zn symmetric cells to achieve stable cycling for more than 360 h under a test condition of 5 mA cm^−2^ and 2 mA h cm^−2^. Further experimental and characterization results demonstrated that the presence of PAA can effectively suppress zinc dendrite formation, mitigate electrode corrosion, and reduce side reactions. These findings confirm that water-soluble polymers can effectively modulate the electrochemical behavior of Zn anode, and they also highlight the application value of PAA as a highly promising additive for environmental stability, cost savings, and long-cycle life in the development of high-performance Zn-based batteries.

## Figures and Tables

**Table 1:** Galvanostatic Zn plating/stripping of long-term cyclic performance of Zn||Zn symmetric batteries at various test conditions.

Test Conditions	Electrolyte System			
	1 M ZnSO_4_	1 M ZnSO_4_ + 0.5 g L^−1^ PAA	1 M ZnSO_4_ + 0.5 g L^−1^ PAM	1 M ZnSO_4_ + 0.5 g L^−1^ PEG
2 mA cm^−2^-2 mA h cm^−2^	65 h	172 h	114 h	166 h
5 mA cm^−2^-2 mA h cm^−2^	107 h	>360 h	300 h	192 h

## Data Availability

The original contributions presented in this study are included in the article. Further inquiries can be directed to the corresponding author.

## Supplementary Materials

The following supporting information can be downloaded at https://www.mdpi.com/article/10.3390/ma18215040/s1, Figure S1: The Coulombic efficiency of Cu||Zn cells in different electrolytes at 5 mA cm^−2^, 1 mAh cm^−2^; Figure S2: FTIR spectrum of Zn-foils that soaked in ZnSO_4_ electrolytes with/without PAA additives for 7 days; Figure S3: SEM images of Zn electrode in Zn||Zn symmetrical batteries after cycling in (a, b) 1 M ZnSO_4_ with 0.5 g L^−1^ PAM electrolytes and (c, d) 1 M ZnSO_4_ with 0.5 g L^−1^ PEG electrolytes; Figure S4: Raman spectra of four electrolytes in the range of 100−700 cm^−1^.
