# Supplementary material for: Stabilizing Zinc Anodes with Water-Soluble Polymers as an Electrolyte Additive
Source: Materials (Basel). 2025 Nov 5;18(21):5040. doi: 10.3390/ma18215040 (PMC12609873; doi:10.3390/ma18215040)
Supplement: Supplementary file 1 [file materials-18-05040-s001.zip › materials-3933639-supplementary.pdf]

Stabilizing zinc anodes with water-soluble polymers as an electrolyte additive

Xueyan Li [1], Xiao-Jiang Chen [2], Senlong Zhang [1], Jinrong Wang [3], Zhuo Chen [4,*], Yue-Xian Song [2,*]

[1] *Shanxi Key Laboratory of Catalysis and Energy Coupling, School of Chemical Engineering and Technology, Taiyuan University of Science and Technology, Taiyuan 030024, China*

[2] *School of energy and power engineering, North University of China, Taiyuan 030051, Shanxi, China*

[3] *Physical Sciences and Engineering Division, King Abdullah University of Science and Technology (KAUST), Thuwal 23955−6900, Saudi Arabi*

[4] *Computer, Electrical and Mathematical Science and Engineering Division, King Abdullah University of Science and Technology (KAUST), Thuwal, 23955−6900, Saudi Arabia*

*Correspondence: zhuo.chen.1@kaust.edu.sa (Z. Chen), songyuexian@nuc.edu.cn (Y.-X. Song)

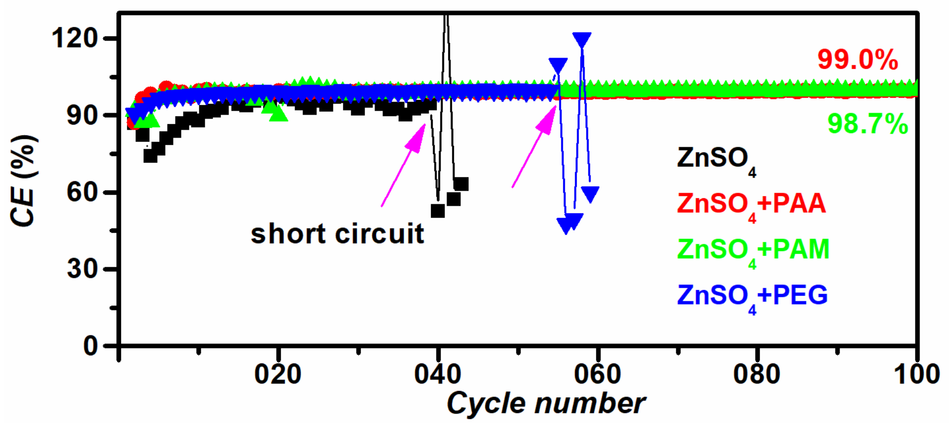

Figure S1 The Coulombic efficiency of Cu||Zn cells in different electrolytes at 5 mA cm^{-2}, 1 mAh cm^{-2}.

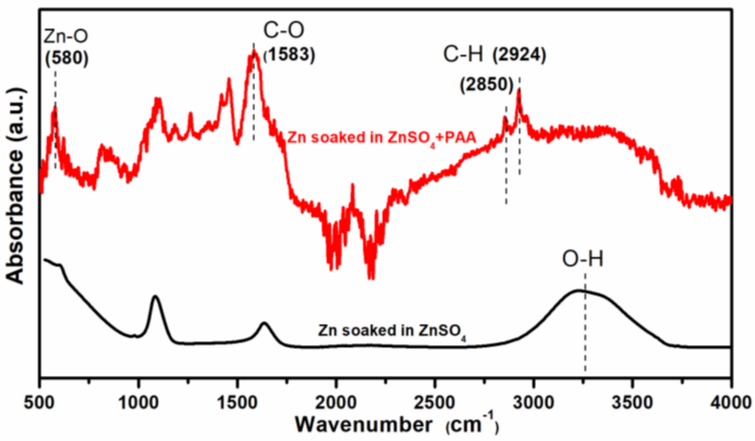

Figure S2. FTIR spectrum of Zn-foils that soaked in $ZnSO_4$ electrolytes with/without PAA additives for 7 days.

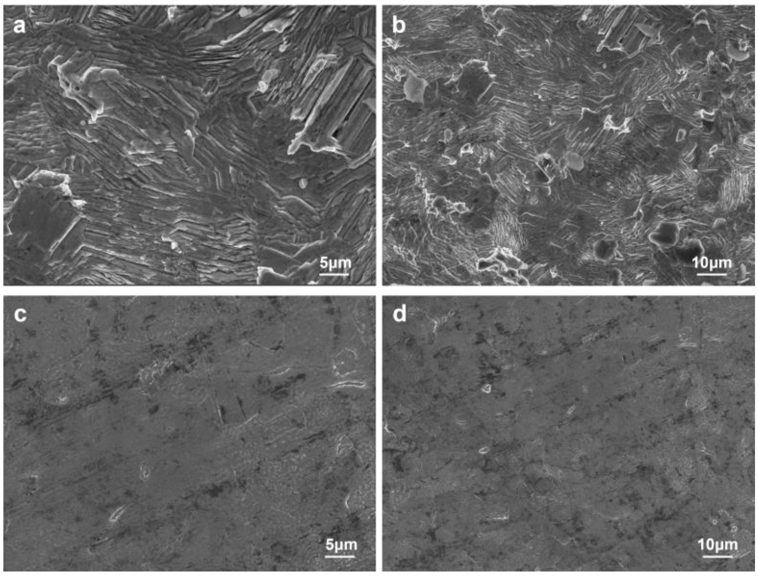

Figure S3. SEM images of Zn electrode in Zn||Zn symmetrical batteries after cycling in (a, b)1 M $ZnSO_4$ with 0.5 g L^{-1} PAM electrolytes and (c, d)1 M $ZnSO_4$ with 0.5 g L^{-1} PEG electrolytes.

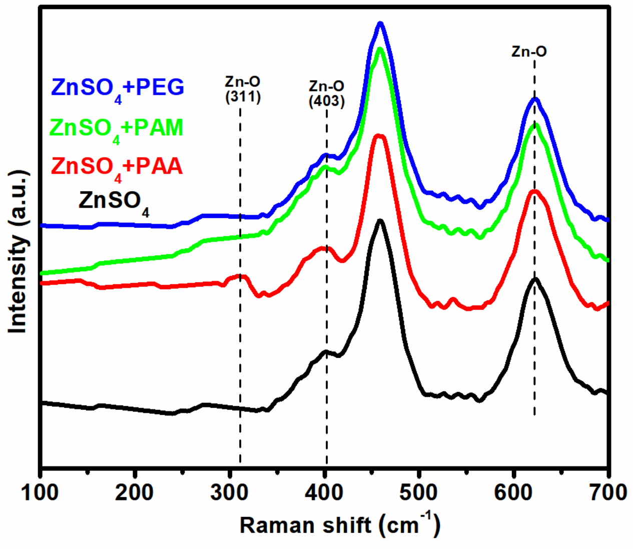

Figure S4. Raman spectra of four electrolytes in the range of 100-700 cm^{-1}.
